# Bradycardia-induced heart failure with preserved ejection fraction successfully treated with empagliflozin and theophylline: a case report

**DOI:** 10.1093/ehjcr/ytae481

**Published:** 2024-09-05

**Authors:** Dino Miric, Marina Juric Paic, Josip Andelo Borovac

**Affiliations:** Cardiovascular Diseases Department, University Hospital of Split, Soltanska 1, 21000 Split, Croatia; Cardiovascular Diseases Department, University Hospital of Split, Soltanska 1, 21000 Split, Croatia; Cardiovascular Diseases Department, University Hospital of Split, Soltanska 1, 21000 Split, Croatia; Department of Pathophysiology, University of Split School of Medicine, Soltanska 2, 21000 Split, Croatia

**Keywords:** Bradyarrhythmia, Bradycardiomyopathy, Case report, Empagliflozin, Heart failure with preserved ejection fraction, HFpEF, SGLT2 inhibitor

## Abstract

**Background:**

The SGLT2 inhibitor empagliflozin has recently gained approval for treating heart failure (HF) across the entire spectrum of ejection fractions including heart failure with preserved ejection fraction (HFpEF). Bradycardia-induced HF, previously described in the literature as bradycardiomyopathy, is an uncommon cause of HFpEF.

**Case summary:**

Herein, we describe a case of a young, 32-year-old woman with no prior medical history who was referred to the hospital due to progressive fatigue and exercise intolerance. She exhibited junctional bradycardia and sinus node dysfunction on electrocardiographic examination, was hypotensive, and had significantly elevated NT-proBNP levels at admission. Transthoracic echocardiographic examination (TTE) revealed preserved systolic function of the left ventricle with segmental abnormalities of contractility and reduced global longitudinal strain, indicative of HFpEF. Cardiac magnetic resonance imaging showed hypertrabeculations, suggesting noncompaction cardiomyopathy (NCCM), even though the definitive diagnostic criteria for NCCM were not met. The patient reported no recent episodes of fever and no chest pain. A comprehensive panel for cardiotropic viruses and Lyme disease were negative while infiltrative diseases such as sarcoidosis were clinically ruled out. Coronary angiography excluded coronary artery disease. Due to profound hypotension and bradycardia, we prescribed empagliflozin and theophylline. At the subsequent follow-up visit within 1 month, the patient reported that she was asymptomatic, with restored sinus rhythm, and complete normalization of NT-proBNP values.

**Discussion:**

Bradycardia-induced HFpEF is a rare entity that can limit the use of most cardiovascular pharmacotherapies but can be successfully treated with empagliflozin and theophylline as demonstrated in our case.

**Learning points**

- Recognize that cardiomyopathy and heart failure with preserved ejection fraction of unclear etiology could be induced by profound bradycardia
- This condition, also known as bradycardiomyopathy, rarely occurs but could be explained by chronic volume overload precipitated by prolonged diastole
- SGLT2 inhibitors such as empagliflozin with the addition of theophylline can be administered to successfully treat HFpEF due to bradycardiomyopathy in patients with profound hypotension and bradycardia

## Introduction

The recent pivotal randomized clinical trials, namely DELIVER and EMPEROR-Preserved, have substantiated the efficacy of SGLT2 inhibitors (dapagliflozin and empagliflozin, respectively) in mitigating mortality and morbidity outcomes among patients with heart failure and preserved ejection fraction (HFpEF).^1,2^ Consequently, guidelines endorse the utilization of SGLT2 inhibitors in HFpEF patients to reduce the risk of heart failure-related hospitalizations and cardiovascular death.^3^ Barriers to full implementation of guideline-directed medical therapy (GDMT), aimed at enhancing outcomes for HF patients, typically involve challenges such as symptomatic hypotension, deteriorating renal function, hyperkalemia or other electrolyte imbalances, and bradycardia.^4,5^ As bradycardia is one of the major points in this case report, it should be acknowledged that SGLT2 inhibitors may offer protection against various forms of arrhythmic disorders.^6^ We present a case of an otherwise healthy 32-year-old female with no pertinent medical history or comorbidities. She presented with progressive fatigue in the context of HFpEF, posing a clinical management challenge due to profound hypotension and bradycardia.

## Summary figure

This figure depicts mechanisms of heart failure with preserved ejection fraction (HFpEF) caused by prolonged diastole due to bradycardia that ultimately led to chronic volume overload and symptoms and signs of heart failure in our patient. Despite having symptomatic hypotension and persistent bradycardia, we instituted treatment with empagliflozin and theophylline which lead to favorable clinical outcomes.

**Figure:**
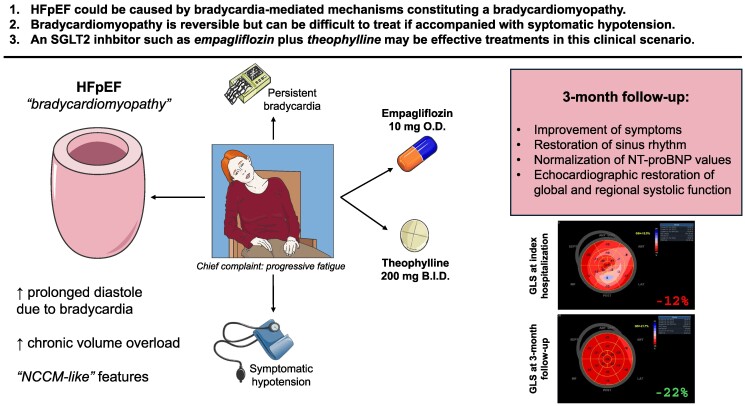


## Case presentation

**Figure 1:**
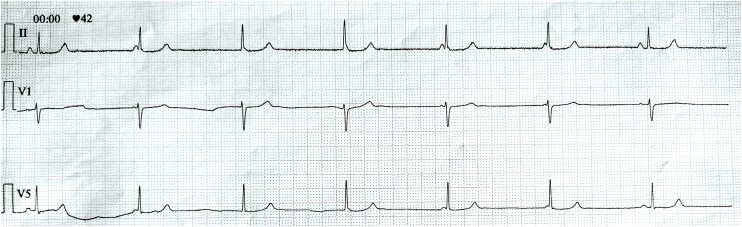
An admission ECG rhythm strip showing sinoatrial delay with junctional rhythm competing with sinus rhythm—the mean heart rate is 42 beats per minute.

A 32-year-old woman presented to the Emergency Department with progressive fatigue and spells of dizziness that were occurring during a period of 2 weeks prior to admission. Fatigue and shortness of breath would occur during ordinary physical activity (NYHA II class). She had no personal or family history significant for cardiovascular disease. She denied any chest pain or syncopal episodes. On physical examination, she had a blood pressure of 70/50 mmHg and bradycardia. An initial electrocardiogram (ECG) revealed a heart rate of 42 beats per minute suggesting sinus node dysfunction and the baseline rhythm of junctional bradycardia (*Figure 1*). Laboratory results showed an NT-proBNP value of 3711 pg/mL (upper reference limit of 300 pg/mL) with high-sensitivity cardiac troponin T (hs-cTnT) of 52.4 ng/L (upper reference limit of 14.0 ng/L). The value of NT-proBNP value obtained by the primary medicine doctor 2 weeks prior was 339 pg/mL (upper reference values of 125 pg/mL for non-acute presentation and 300 pg/mL for acute presentation). The value of C-reactive protein was under the lower diagnostic threshold of <0.6 mg/L (upper reference limit of 5 mg/L). She denied any recent febrility or infectious illnesses.

**Figure 2:**
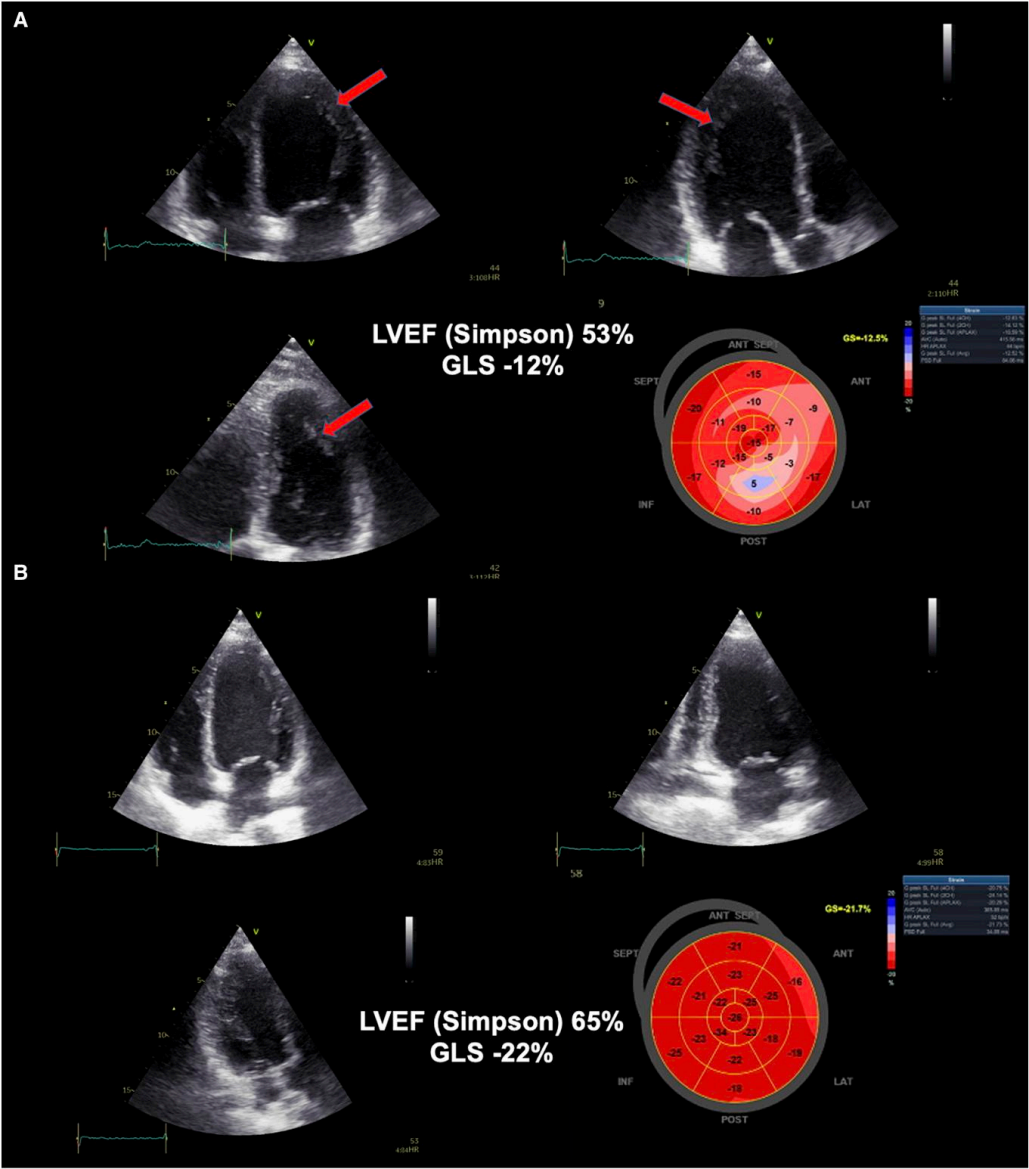
A transthoracic echocardiography examination at (*A*) index hospitalization and (*B*) follow-up examination at 1 month following discharge. The image depicts 2-chamber and 4-chamber views of the heart with ‘bullseye’ plot showing global longitudinal strain values. Red arrows show trabeculation within LV structure.

A serological panel for cardiotropic viruses (including Coxsackie virus, adenovirus, cytomegalovirus, parvovirus B19, human herpesvirus 6, Epstein-Barr virus, herpes simplex virus, hepatitis C, HIV, influenza A, and SARS-CoV-2 virus) and for Lyme disease were performed and were negative. There was a low clinical suspicion for sarcoidosis, given the patient's presentation; therefore, no work-up for sarcoidosis was pursued. A transthoracic echocardiographic (TTE) examination was undertaken and showed a preserved left-ventricular ejection fraction (LVEF) of 53% and significantly reduced global longitudinal strain (GLS) of −12%, especially in the posterolateral and anterior myocardial regions (*Figure 2A*, Supplementary material online, *Video S1*). A coronary angiography revealed patent epicardial coronary vessels with no obstructive atherosclerotic disease (see Supplementary material online, *Videos S2* and *S3*). The left ventriculography showed hypercontractility of basal segments, hypokinesia of mid-LV segments with normal values of left-ventricular end-diastolic gradient, and no valvular dysfunction (see Supplementary material online, *Video S4*), and this did not resemble typical Takotsubo syndrome pattern while patient also denied any psychological or physical triggers and had no history of psychiatric or neurologic disorders.^7^ Furthermore, according to the InterTAK diagnostic score, our patient obtained 31 points yielding the probability of Takotsubo syndrome of only 0.8%.^8^

**Figure 3:**
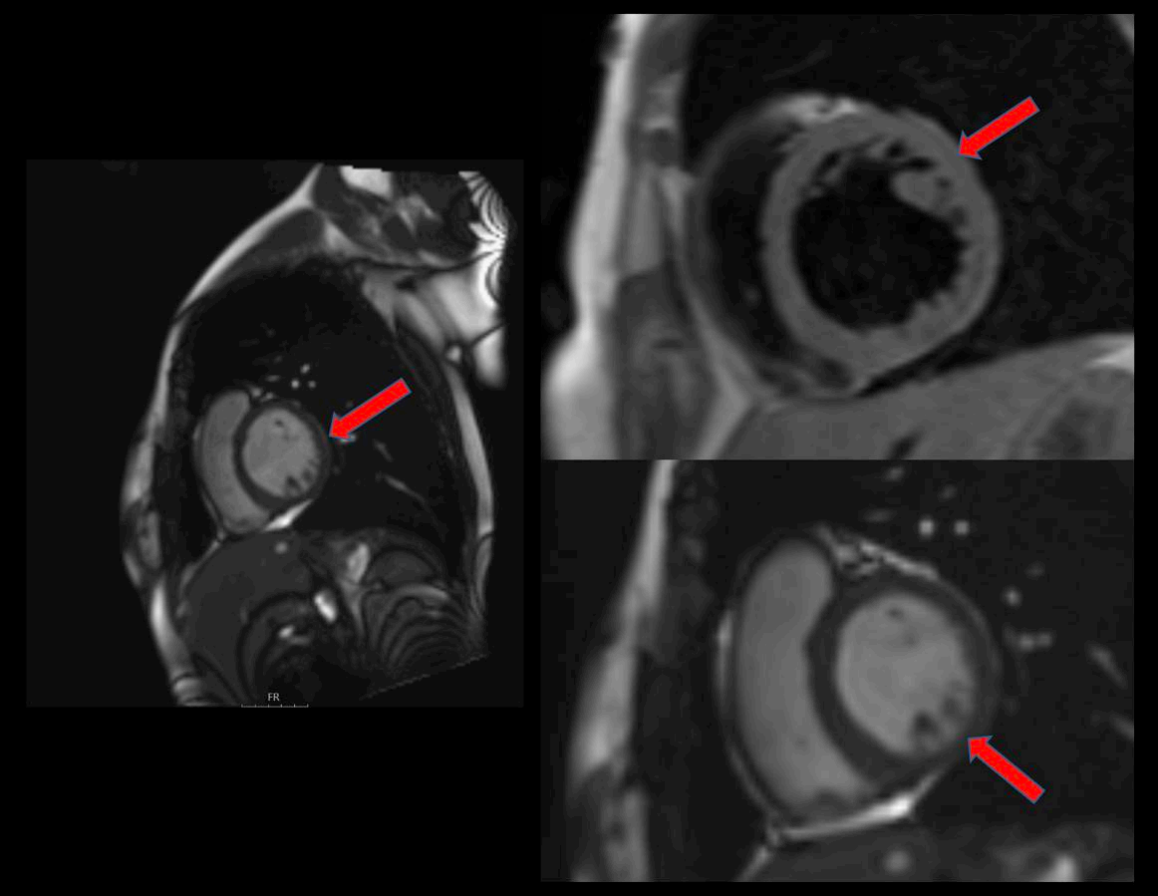
A cardiac magnetic resonance imaging showing the relationship of non-compacted to compacted myocardium up to 2.8 with enhanced trabeculations in the apical third of LV (arrows) with no signs of mural fibrosis.

A patient subsequently underwent cardiac magnetic resonance (CMR) imaging. This showed trabeculations with left-ventricular internal diastolic diameter (LVIDd) of 56 mm and the ratio of noncompacted to compacted myocardium of 2.8 at end-diastole, suggesting the possibility of noncompaction cardiomyopathy (NCCM) (*Figure 3*, Supplementary material online, *Video S5*). Furthermore, CMR-derived LVEF was calculated to be 46% and previously described regional wall motion abnormalities were present. There were no signs of mural fibrosis and the myocardium exhibited normal signal intensities with normal resting myocardial perfusion. However, the patient did not satisfy all CMR imaging criteria for NCCM,^9,10^ and the possibility of pseudo-NCCM due to bradycardia-mediated chronic LV overload was considered.

On day 3 of hospitalization, she experienced restoration of sinus rhythm with T-wave inversion and prolonged corrected QT (QTc) interval calculated by Bazett formula to be 477 milliseconds (Fridericia, 506 msec; Hodges, 538 msec). She showed no signs of clinical congestion; however, she was hypotensive, bradycardic, and had prolonged QTc interval which limited potential treatment options. GDMT therapies such as sacubitril/valsartan, ACE-inhibitors, or other anti-remodeling agents could not be initiated due to hypotension and diuretics would also likely cause volume depletion and further drop in blood pressure. For these reasons and due to the diagnosis of HFpEF we decided to treat her with 10 mg of empagliflozin OD along with theophyilline 200 mg twice-daily (BID) to stimulate sinus node function and this constituted the main therapy at discharge.^3,11^

**Figure 4:**
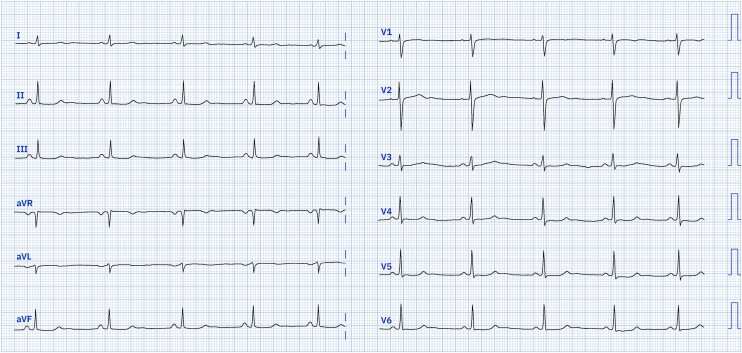
A 12-lead electrocardiogram recorded during the follow-up hospitalization at 1 month following index hospitalization showing sinus rhythm and normal QT interval.

At the 3-month follow-up, she was clinically reevaluated. She reported feeling well with no signs and symptoms of HF at clinical examination. This time, TTE examination showed complete restoration of left-ventricular function with LVEF of 65%, GLS of −22%, and no wall motion abnormalities (*Figure 2B*, Supplementary material online, *Video S6*). Her 12-lead ECG showed a sinus rhythm of 57/min with a Bazett-corrected QT interval of 443 milliseconds (*Figure 4*). Her mean blood pressure taken during hospitalization was stable at a mean of 110/70 mmHg, and she experienced no episodes of hypotension or dizziness. Moreover, the NT-proBNP value significantly decreased to 158 pg/mL (upper reference limit, 300 pg/mL) while hs-cTnT was 5.0 ng/L (upper reference limit, 14.0 ng/L). Finally, her chronotropic competence was confirmed with successful treadmill stress testing according to Bruce's protocol.

## Discussion

This case highlights some of the unique challenges when dealing with a young patient who presented with signs and symptoms of HFpEF of unclear etiology. Our patient had bradycardia along with sinus node dysfunction and signs of sinoatrial delay that likely contributed to heart failure with dissociation of the systolic and diastolic cycle. As can be appreciated from the initial ECG, the ventricular rate was stable following a junctional pattern whereas the sinus node is slow and resets (*P*-wave morphology is shown independently while in some instances buried within the QRS complex). Furthermore, she presented with symptomatic hypotension and also exhibited signs of long QT on ECG that prevented the use of anti-remodeling agents such as ACE-inhibitors or mineralocorticoid receptor antagonists (MRAs)s and other GDMT such as beta-blockers. Instead, we administered an SGLT-2 inhibitor, empagliflozin, in a dose of 10 mg OD along with theophylline 200 mg BID in order to improve sinus node function. It has been previously shown that theophylline exerts a positive chronotropic effect, and this is likely achieved through its antagonism of cardiac effects of adenosine that is known for its depression of sinus node automaticity.^12^ This treatment finally resulted in a marked improvement in the patient's symptoms, significantly reduced circulating natriuretic peptide concentrations, and restored normal global and regional systolic function, at the 3-month follow-up.

The course of the patient presentation and worsening of symptoms seemed to be subacute in this patient (period of 2 weeks) with a significant rise in natriuretic peptides between the 2 time points. Moreover, the etiology of HF in the presented case was unclear as the patient had no pertinent history of cardiovascular disease and no identifiable risk factors. Viral etiology was ruled out since the panel for cardiotropic viruses was negative and Takotsubo syndrome was clinically excluded. While the patient did exhibit left-ventricular hypertrabeculation according to CMR and TTE imaging, the criteria for NCCM were not fulfilled.^9,10^ In fact, recent ESC guidelines on cardiomyopathy do not consider NCCM as a cardiomyopathy but rather refer to it as a phenotypic trait that occurs either in isolation or in tandem with other developmental abnormalities, systolic dysfunction, dilatation, or LV hypertrophy.^13^ Infiltrative diseases such as sarcoidosis and infectious causes such as Lyme carditis might also act as possible etiologies of bradycardia and atrioventricular (AV) node disturbances and should be taken into account when examining patients with HF of unclear origin. Perhaps, the patient's symptoms of HF and resemblance to NCCM might be explained by the profound bradycardia as this would sustain chronic volume overload enhanced by prolonged diastole while Caliskan and colleagues even coined a term for this entity—bradycardiomyopathy.^14^ Sinus bradycardia is often associated with NCCM due to the postulated theory that the development of NCCM may include myocardial vascular abnormality in the blood supply of the sinus node during embryonic development and cardiac angiogenesis.^15^ A genetic testing for LV noncompaction can also be considered although the diagnostic yield of this work-up in adults appears to be low, especially in cases of isolated suspicion of NCCM in patients without a family history.^16^ It should also be noted that there are pathologic *HCN4* gene variants that are associated with bradycardic forms of left-ventricular noncompaction cardiomyopathy.^17^ Finally, the presence of intra-atrial delay on initial ECG such as appreciated in this case might prompt further electrophysiological study (EPS) as this might be caused by atrial fibrosis not captured on CMR. This could further refine treatment strategies, for example, potential pacemaker implantation if symptoms persisted and if the patient did not clinically improve. However, due to a patient young age and the potential to improve with pharmacotherapy, we opted for a conservative approach.

## Conclusions

Bradycardia-related conduction disorders could be suspected as a culprit for HFpEF development among patients with unclear etiology of HF. In such patients who also present with symptomatic profound hypotension, the use of HF-directed pharmacotherapies might be limited. Empagliflozin, with an addition of theophylline, was successful in treating bradycardiomyopathy (Summary Figure). Furthermore, the electrophysiological study might be considered due to the evidence of initial intra-atrial delay and this might help in refining treatment strategies such as pacemaker implantation if symptoms persisted or if there was no clinical improvement due to a failure of a conservative medical approach.

## Lead author biography


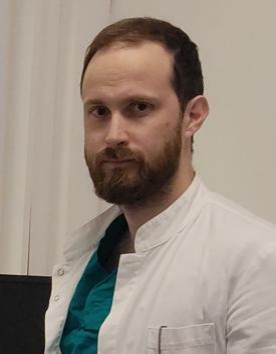


Dr. Dino Miric (MD, PhD) is an interventional cardiologist at the Cardiovascular Diseases Department, University Hospital of Split in Split, Croaetia. His areas of scientific and professional interest are coronary artery disease, complex percutaneous coronary interventions, and heart failure.

## Supplementary Material

ytae481_Supplementary_Data

## Data Availability

The data underlying this article will be shared on reasonable request to the corresponding author.

## Notes

**Consent:** The authors confirm that written consent for submission and publication of this case report including the images and associated text has been obtained from the patient in line with COPE guidance.

**Funding:** Information used for this manuscript was collected during project of Boehringer Ingelheim - NTA 22-100725. The author/s would like to acknowledge the financial support from Boehringer Ingelheim, which covered the submission fee for this journal. This funding has not influenced the design of manuscript, execution, interpretation, or decision to submit the work for publication. Boehringer Ingelheim was given the opportunity to review the manuscript for medical and scientific accuracy as it relates to Boehringer Ingelheim substances, as well as intellectual property considerations.
